# Influence of voluntary action and outcome valence on the sense of agency

**DOI:** 10.3389/fnhum.2023.1206858

**Published:** 2023-09-07

**Authors:** Nana Niu, Yun Wu, Hui'e Li, Mei Li, Danping Yang, Wei Fan, Yiping Zhong

**Affiliations:** ^1^Department of Psychology, Hunan Normal University, Changsha, China; ^2^Cognition and Human Behavior Key Laboratory of Hunan Province, Changsha, China; ^3^Institute of Interdisciplinary Studies, Hunan Normal University, Changsha, China

**Keywords:** voluntary action, outcome valence, sense of agency, cognitive process, N1, N300

## Abstract

Recent studies have revealed that people might experience a lessened sense of agency for negative consequences by claiming that they were obeying orders. However, little is known about the cognitive neural mechanism behind the reduced sense of agency when individuals are forced to inflict physical harm on others. This study adopted temporal estimation tasks to investigate the internal mechanism of voluntary action on the sense of agency and the moderating effect of outcome valence as measured by event-related potentials (ERPs). In the temporal estimation task, participants were asked to make trade-offs of monetary gains for themselves against painful electric stimuli experienced by strangers, subjectively estimated the perceptual temporal interval between keypress actions (i.e., free or coercive actions) and consequent outcomes (i.e., positive or negative tones) and rated the feeling of control. The results showed that perceived temporal interval was shorter for positive tones compared with negative tones in the coercive condition, and induced more negative N1 and N300 amplitudes, which indicated that the implicit sense of agency was higher. However, the explicit sense of agency was stronger in the free condition than in the coercive condition, which was not influenced by outcome valence. We discuss the implications of utilizing positive feedback and free choice as significant strategies for those experiencing the abnormal sense of agency.

## 1. Introduction

Sense of agency refers to the subjective experience of controlling one's actions and, through them, external events (Haggard et al., 2002; Caspar et al., 2016; Haggard, 2017). For example, “I” am the person who caused something to move (Li, 2016), which is the core element that makes individuals responsible for their behavior to foster social cohesion (Sun et al., 2023). When individuals perceive that they have initiated behavior, they can take the initiative to control and coordinate their behavior to achieve established goals (Haggard, 2017). However, studies have demonstrated that individuals who follow orders to harm others tend to feel less responsible for their actions (Milgram, 1963), attenuating the sense of agency (Caspar et al., 2016, 2018). However, few studies have explored the internal mechanism of the reduced sense of agency and how to moderate the subjective experience when forced to inflict harm upon others. Given that the abnormal sense of agency is associated with serious mental illnesses (Blakemore et al., 2000), this study explored the internal mechanism of the reduced sense of agency in moral dilemmas, which will help further understand the embodied psychological mechanism across different situations.

Sense of agency is a multi-faced experience that comprises implicit and explicit components (David et al., 2008; Sun et al., 2023). The implicit measure is the temporal estimation for intentional binding, which asks individuals to estimate the temporal interval between actions and consequent outcomes (Buehner and Humphreys, 2009; Humphreys and Buehner, 2010; Caspar et al., 2016; Imaizumi and Tanno, 2019; Malik and Obhi, 2019; Suzuki et al., 2019; Sun et al., 2023). The perceived shorter temporal interval between keypress actions and consequent outcomes reflects stronger implicit sense of agency (Haggard et al., 2002; Wenke and Haggard, 2009; Barlas et al., 2017; Bu et al., 2022; Huang et al., 2023), which involves “binding” consequent outcomes (e.g., tones) with self-actions (Sun et al., 2023). The explicit sense of agency is generally subjectively rated as the extent that individuals feel in control of their actions and the resulting outcomes (Hoogeveen et al., 2018; Suzuki et al., 2019; Sun et al., 2023).

Voluntary action is one of the important factors that influence the sense of agency. Individuals make actions based on free will, which subsequently result in corresponding outcomes. The greater the degree of voluntary action, the greater the sense of agency (Bu et al., 2022). For example, Tanaka and Kawabata (2021) manipulated free choice (i.e., chose one of eight keys) and no-choice (i.e., specific one instructed), which showed that the sense of agency is higher during free choice (Tanaka and Kawabata, 2021). Contrastingly, several studies have suggested that the sense of agency is reduced when individuals are forced to inflict electric stimuli on strangers (Caspar et al., 2016, 2018). Why do individuals experience inconsistent sense of agency in different contexts? Prospective accounts indicate that the sense of agency arises before the outcome occurs, that is, the choice itself induces the sense of agency (Wegner and Wheatley, 1999; Wolpert and Ghahramani, 2000; Haggard and Clark, 2003; Engbert et al., 2007; Yoshie and Haggard, 2017). Specifically, when faced with two switches, if the intended action is to choose the lighting switch, one will experience the sense of agency over the action; however, if the decision to choose the switch is hesitant, the sense of agency will be relatively weakened (Li, 2016). According to prospective accounts, we speculated that being forced to inflict physical harm on others could provide them with a suitable excuse to evade responsibility, consequently leading to a diminished sense of agency in the coercive condition.

Notably, a few studies within the field of sense of agency have discovered the importance of outcome valence. These studies have demonstrated that negative outcomes weaken the experience of sense of agency (Takahata et al., 2012). Specifically, the sense of agency reduces self-actions in negative outcomes compared with either positive or neutral outcomes (Gentsch et al., 2015; Christensen et al., 2016, 2019; Yoshie and Haggard, 2017; Barlas et al., 2018). This reduction in the sense of agency may be attributed to an individual's self-serving bias, tending to attribute success to themselves and failure to external events (Bu et al., 2022). The above findings are inconsistent with prospective accounts where the sense of agency arises before the outcome occurs. Based on the existing studies, there could be other underlying mechanisms involved in the sense of agency.

Retrospective accounts show that the sense of agency is inferred from the causal relationship between action and consequent outcome (Wegner, 2003; Moore and Haggard, 2008; Moore and Obhi, 2012). According to retrospective accounts, the sense of agency is generated when the outcome is consistent with actual action. The higher the consistency, the stronger the sense of agency. For example, studies have demonstrated that retrospective inference plays an important role in intentional binding (Moore and Haggard, 2008). The more severe the immoral outcomes, the stronger the implicit sense of agency, which could be attributed to the retrospective enhancement of causal associations between actions and subsequent outcomes through the sense of guilt (Moretto et al., 2011; Bu et al., 2022). Therefore, when negative outcomes occur, the decreased sense of agency could be owing to the retrospective enhancement of the causal connection between actions and the negative outcomes. On the basis of these findings, this study employed event-related potentials (ERPs) to further provide cognitive and neural evidence for the underlying processing mechanisms of the sense of agency.

Event-related potential (ERP) is a high-temporal resolution technique to examine the temporal dynamics of brain responses associated with the sense of agency. Generally, the underlying neural basis of how voluntary action and outcome valence influence the sense of agency in unethical contexts remains unclear. Studies have demonstrated that the early attention-related ERP component is observed only when participants believe that the behavior is controlled by themselves (Ciardo et al., 2020). Self-generated auditory (e.g., tones) or visual stimuli (e.g., pictures) elicit a weaker N1 component compared with externally initiated stimuli, indicating the presence of sensory attenuation (Schafer and Marcus, 1973; Gentsch et al., 2015). This sensory attenuation was observed regardless of whether the tones were predictable or not via self-generation (Bäß et al., 2008). However, there is no significant difference in the N1 component between self-initiated actions and externally-initiated actions, discovering that more negative N1 is elicited in incongruent tones compared with congruent tones (Kühn et al., 2011). The aforementioned studies mainly focused on the sensory attenuation of visual or auditory stimuli concerning self-other distinctions, without exploring the temporal dynamics of how voluntary action and outcome valence influence the sense of agency in unethical contexts. When individuals are free to inflict electric stimuli on others, the neutral tones elicit more negative N1, which reflects the causal relationship between self-actions and consequent outcomes (Caspar et al., 2016). Considering the aforementioned divergent research outcomes, we further investigated whether individuals experience sensory attenuation or enhancement when they are acting freely or under coercion to inflict physical harm on others.

In addition, the N300 component has been linked to the affective evaluation of stimuli (Carretié and Iglesias, 1995; Rossignol et al., 2005; Ruz et al., 2013), a negative deflection peaking ~300 ms, which is supposed to reflect the depth of affective processing or the affective significance of stimuli rather than the physical characteristics (Rossignol et al., 2005; Ruz et al., 2013). For example, researchers have investigated how individuals process facial expressions of different valences and found that angry facial expressions elicit more negative N300, which is associated with the processing of emotional valence (Schutter et al., 2004). Similarly, angry facial expressions elicit more negative N300 (Carretié and Iglesias, 1995; Carretié et al., 1997; Rossignol et al., 2005). Furthermore, researchers have, for the first time, investigated the temporal dynamics of affective modulation of the sense of agency in self-generated and externally generated outcomes, indicating that self-generated visual stimuli elicit more negative N300 compared with externally initiated stimuli, and that self-generated negative outcomes evoke more negative N300 compared with self-generated positive tones during later stages of cognitive processing (Gentsch et al., 2015). Consequently, we speculated that individuals, influenced by self-serving bias, tend to associate positive outcomes with themselves for self-enhancement, while negative outcomes are more commonly attributed to others. Given that few studies have explored the temporal dynamics and affective processing of the reduced sense of agency, this study aimed to shed more light on the neural correlates of the interaction between voluntary action and outcome valence on the sense of agency when individuals are free or forced to inflict physical harm on others.

To summarize, this study adopted the temporal estimation task to investigate the neurocognitive mechanisms of how outcome valence moderated the reduced sense of agency. We hypothesized that participants might experience a stronger sense of agency when provided with positive outcomes in the coercive condition, which would be reflected in the ERP activation patterns. Specifically, as the N1 and N300 components reflect the causal relationship between actions and consequent outcomes and the effective processing of outcome valence respectively, positive outcome would elicit more negative N1 and N300 in the coercive condition compared with the coercive-negative condition.

## 2. Methods

### 2.1. Participants

To mitigate the influence of individual differences in time interval perception and feeling of control, we employed a within-subject experimental design. A power analysis using G^*^Power 3.1 (Faul et al., 2007) indicated that 24 participants would ensure 80% statistical power and even in the case of medium effect size (i.e., *F*-test for repeated measures ANOVA with within-factors, *f* = 0.25). Considering participant attrition and skepticism regarding the authenticity of the experiment, we recruited 36 participants from Hunan Normal University using random sampling, specifically by posting posters on campus. The data from 27 participants (15 men; *M* = 21.22 years, *SD* = 2.15 years) were ultimately used for analysis. All participants were right-handed, had normal or corrected vision, and had no neurological or psychiatric history. Informed consent was obtained, and participants were informed that they had the right to freely withdraw from the experiment. Our research was conducted in accordance with the Declaration of Helsinki and approved by the Ethics Committee of Hunan Normal University.

After the experiment, participants were questioned regarding their belief in the administration of real electric stimulation, and those expressing skepticism about delivering electric shocks were excluded from further analysis. Furthermore, it was explained to participants that the formal experiment did not inflict electric stimuli on others to ensure there was no substantive injury to participants, and the corresponding experimental reward was paid.

### 2.2. Materials

#### 2.2.1. Painful electric stimuli

Overall, 1 week before the experiment began, 30 participants (15 men) were selected to complete the pain stimulus assessment adapted from the study by Zhan et al. (2020), who were independent of the formal experiment. The participants were asked to use the multichannel electrical stimulator to deliver a series of gradually increasing painful electric stimuli from 0.1 to 9 mA and rated the experience of pain on an 11-point scale ranging from 0 (no pain) to 10 (intolerable) (Caspar et al., 2016; Christensen et al., 2019; Zhan et al., 2020). The one-sample *t*-test was conducted for the pain rating. Compared with the median 5, the results indicated no significant difference in the intensity of electrical stimuli, *t*_(29)_ = 1.71, *p* = 0.10. At 6 mA, there was a significant difference, *t*_(29)_ = 4.34, *p* < 0.001, Cohen's *d* = 3.42. To create a moral dilemma, the formal experiment selected the 6–9 mA electric stimuli range.

#### 2.2.2. The manipulation of voluntary action

Following the procedure used in previous studies (Caspar et al., 2016, 2017, 2018), this study manipulated the level of voluntary action by allowing participants to freely choose or comply with electric shock commands, potentially resulting in physical harm to others, with a monetary benefit of 0.5 Yuan per electric stimulation. Specifically, in the free condition, participants were given the freedom to determine whether to administer electric stimuli to a stranger for personal financial gain. In the coercive condition, the computer randomly presented a red frame in half of the coercive condition (30/60 times), and participants were forced to inflict electric stimuli on a stranger when the red frame appeared (Caspar et al., 2016). It should be noted that failure to comply with the commands led to the termination of the experiment.

#### 2.2.3. Outcome valence stimuli

Overall, 1 week before the experiment began, we selected 20 positive and 20 negative tones from the International Affective Digitized Sounds (Bradley and Lang, 2007; Tanaka and Kawabata, 2021) and recruited 30 participants to rate the degree of emotional valence and arousal on a seven-point scale, ranging from 1 (strongly disagree) to 7 (strongly agree), who were independent of the formal experiment. Finally, we selected four highly emotionally negative tones and four highly emotionally positive tones which were all trimmed to 700 ms and had intensity standardized. The average scores of negative and positive tones were tested with the paired sample *t*-test. The results showed that the valence of positive (*M* = 4.60, *SD* = 1.18) and negative tones (*M* = 2.16, *SD* = 0.80) was significantly different, *t*_(29)_ = 10.25, *p* < 0.001, Cohen's *d* = 2.42. Regarding arousal, there was no significant difference between positive (*M* = 4.61, *SD* = 1.20) and negative tones (*M* = 4.27, *SD* = 0.90), *t*_(29)_ = 1.77, *p* > 0.05.

### 2.3. Experimental design

A 2 (voluntary action: free, coercion) × 2 (outcome valence: positive, negative) within-subject experimental design was employed.

### 2.4. Procedure

The experiment consisted of three stages. The first was the pain threshold measured before the experiment. Participants sat comfortably in a room ~75 cm away from a 15-inch (~38 cm) color computer screen. Two electrodes connected to the stimulator were placed on the participants' left hands, which delivered a series of gradually increasing painful electric shocks from 0.1 to 9 mA. Subsequently, participants were informed of their pain threshold and, importantly, the intensity of electric stimuli (i.e., 6–9 mA) inflicted on a stranger in the formal experiment by themselves, which created a moral conflict.

Subsequently, participants engaged in the practice session to enhance their perception of the temporal interval, aiming to improve their ability to accurately perceive and discriminate time delays. Specifically, participants were asked to press one button (i.e., the “F” key or the “J” key), which made a neutral tone after a random delay ranging from 100 ms to 1,000 ms. Importantly, participants were asked to input the perceptual temporal interval between keypress actions and consequent tones. Then, the computer presented the correct temporal interval. The practice session ended after 12 trials. If participants were unable to accurately perceive the temporal interval, the exercise was repeated.

Finally, the experiment commenced. The electrodes were relocated from the participants' left hand to that of a same-sex stranger (i.e., experimental assistant, woman–woman, or man–man), who sat in the adjacent room visible to participants. During the formal experiment, first, a fixation point was presented for 500 ms. Then, a decision interface appeared, and participants were instructed to press the “F” key (i.e., deliver the electric shock stimulus) or the “J” key (i.e., decline to deliver the electric shock stimulus). Subsequently, the computer presented a positive or negative tone in the free and coercive conditions, respectively. The delay between the keypress actions and the subsequent tones was randomly varied to intervals of 200 ms, 500 ms, and 800 ms. Finally, participants were asked to input a three-digit number to indicate their perceived time interval between keypress actions and subsequent tones, with a range from 1 ms to 1,000 ms.

**Figure 1:**
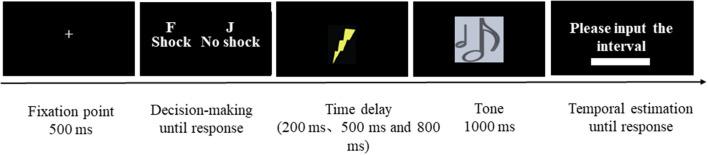
Schematic representation of a single trial. Initially, a fixation point appeared at the center of the screen, reminding participants to focus their attention. After a duration of 500 ms, the fixation point disappeared. Following that, the decision-making interface appeared, where participants were instructed to press “F” to inflict harm on strangers and earn 0.5 Yuan or press “J” to refrain from inflicting harm without receiving any benefit. Subsequently, tones occurred with time delays of 200 ms, 500 ms, or 800 ms. Finally, participants were asked to input a three-digit number to indicate their perceived time interval between keypress actions and subsequent tones, with a range from 1 to 1,000 ms.

To summarize, the experiment consisted of four blocks (coercive-positive, coercive-negative, free-positive, and free-negative), which were balanced between participants. Each block had 60 trials. The sequence of every trial is presented in Figure 1. After each block, participants were asked to answer the question “To what extent do you think you have control over behavioral outcomes?” on a seven-point scale, in which 1 represented “not at all” and 7 represented “complete control” (Caspar et al., 2016).

### 2.5. EEG recording and processing

The electroencephalogram (EEG) was recorded from 64 Ag/AgCl electrodes mounted in an elastic cap, according to the International 10/20 EEG/ERP System (ANT Neuro, Enschede, the Netherlands). The EEG recording was continuously sampled at 500 Hz with CPZ and referenced offline using the average of the left and right mastoids. The impedance at all recording sites was < 5 kΩ. For offline analysis, data were preprocessed using MATLAB 2013a (Delorme and Makeig, 2004; Plöchl et al., 2012; Zhan et al., 2020), which was filtered with a 0.1 to 30-Hz bandpass filter. Independent component analysis was used to remove blinking and artifacts (Delorme and Makeig, 2004). Trials in which EEG voltages exceeded the threshold of ±80 μV were excluded from the analysis (Zhan et al., 2020). After excluding outliers, the average number of trials for each experimental condition remained above 96%. Specifically, the average number of trials was as follows: 59.19 for the free-positive condition, 58.60 for the coercion-positive condition, 58.41 for the free-negative condition, and 57.78 for the coercion-negative condition. Epochs were extracted from 200 ms before to 600 ms after the tone presentation. Activity in the −200 ms to 0 ms time-window prior to the tone interface served as the baseline for each ERP (Zhan et al., 2018, 2020; Li et al., 2021; Fan et al., 2022).

### 2.6. Data analyses

For behavioral data, the repeated-measures analysis of variance [ANOVA; 2 (voluntary action: free, coercion) × 2 (outcome valence: positive, negative)] was performed by comparing each dependent variable (temporal interval, feeling of control). The *p*-values for main and interaction effects were corrected by the Greenhouse–Geisser method for violations of the sphericity assumption, and Bonferroni corrections were used for multiple comparisons.

For ERP data, combined with previous ERP studies (Schafer and Marcus, 1973; Rossignol et al., 2005; Bäß et al., 2008; Gentsch et al., 2015; Caspar et al., 2016) and visual observation for the brain topographic map (see **Figure 3**), 6 electrodes (P3, Pz, P4, PO3, POz, and PO4) were selected for the N1 component (70–200 ms) and 15 electrodes (F3, Fz, F4, FC3, FCZ, FC4, C3, CZ, C4, CP3, CPz, CP4, P3, Pz, and P4) were selected for the N300 component (250–400 ms). ERP data were measured as the mean amplitudes for each brain region. Repeated-measures ANOVA was conducted using the following factors: (voluntary level: free, coercive) × (outcome valence: positive, negative) × (Caudality: N1: parietal, parietal-occipital; N300: frontal, frontal-central, central, central-parietal, and parietal).

Pearson's correlation analysis was further used to measure the correlation between the implicit and explicit sense of agency.

## 3. Results

### 3.1. Manipulation check

The results revealed that the proportion of electric stimuli selected was 48% 95% CI = (32%, 63%) in the free-positive condition; 41%, 95% CI = (26%, 56%) in the free-negative condition; 67%, 95% CI = (59%, 75%) in coercive-positive condition; and 64%, 95% CI = (55%, 72%) in coercive-negative condition. The repeated-measures ANOVA of the proportion of electric stimuli showed that the main effect of voluntary action was significant, *F*_(1,26)_ = 18.29, *p* < 0.001, $\eta_{p}^{2}$ = 0.42, indicating that the proportion of electric stimuli in the coercive condition was significantly higher than that in the free condition. No significant difference was found in other main effects and interactions, *ps* > 0.05, indicating that the manipulation of free and coercive conditions was effective.

### 3.2. Behavior results

#### 3.2.1. Implicit sense of agency

**Figure 2:**
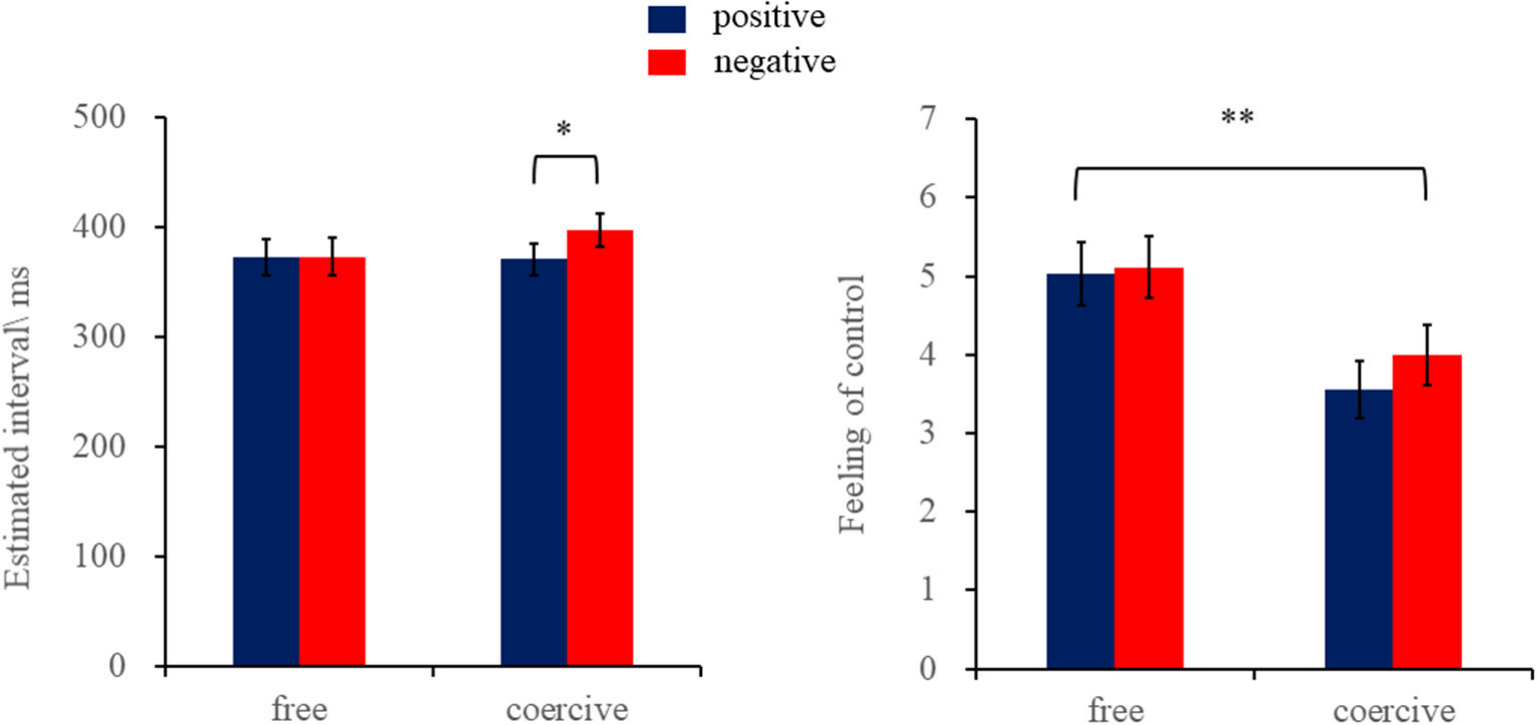
The estimated temporal intervals **(left)** and feeling of control **(right)** under different conditions. *p < 0.05.

The results indicated that the main effect of voluntary action was not significant, *F*_(1,26)_ = 2.22, *p* = 0.15, and $\eta_{p}^{2}$ = 0.08. Moreover, the main effect of outcome valence was not significant, *F*_(1,26)_ = 2.07, *p* = 0.16, and $\eta_{p}^{2}$ = 0.07. There was a significant interaction of voluntary action and outcome valence, *F*_(1,26)_ = 4.82, *p* < 0.05, and $\eta_{p}^{2}$ = 0.16. Furthermore, the simple effect analysis revealed that the estimated temporal interval in the coercive-positive condition (*M* = 370.78 ms, *SD* = 14.26 ms) was shorter compared with the coercive-negative condition (*M* = 397.27 ms, *SD* = 15.37 ms, *p* < 0.05), indicating a stronger sense of agency in the former condition. However, in the free condition, the interval estimates between keypress actions and positive tones (*M* = 372.41 ms, *SD* = 16.89 ms) were not significantly different from those of negative tones (*M* = 372.92 ms, *SD* = 17.15 ms, *p* = 0.97; see Figure 2).

#### 3.2.2. Explicit sense of agency

The results indicated that the main effect of voluntary action was significant, *F*_(1,26)_ = 12.87, *p* < 0.01, and $\eta_{p}^{2}$ = 0.33, indicating that the feeling of control was lower in the coercive condition (*M* = 3.78, *SD* = 0.34) as compared with the free condition (*M* = 5.07, *SD* = 0.38). In addition, the main effect of outcome valence and the interaction between voluntary action and outcome valence were not significant, *ps* > 0.05 (see Figure 2).

The correlation between the implicit and explicit sense of agency did not reach the level of statistical significance, *ps* > 0.05. Moreover, we further adopted a regression analysis to analyze the independent prediction effect of electric stimuli proportion in four situations (free-positive, free-negative, coercive-positive, and coercive-negative) on the sense of agency, aiming to exclude the influence of electric stimuli proportion on the implicit and explicit sense of agency. The results showed that the electric stimuli proportion had no significant predictive effect on the estimated temporal interval (*bs* < 0.16, *ps* > 0.05) or the feeling of control (*bs* < 0.33, *ps* > 0.05), which were consistent with the finding by Caspar et al. (2016) that the proportion of electric stimuli did not significantly affect the sense of agency.

### 3.3. ERP results

#### 3.3.1. N1

**Figure 3:**
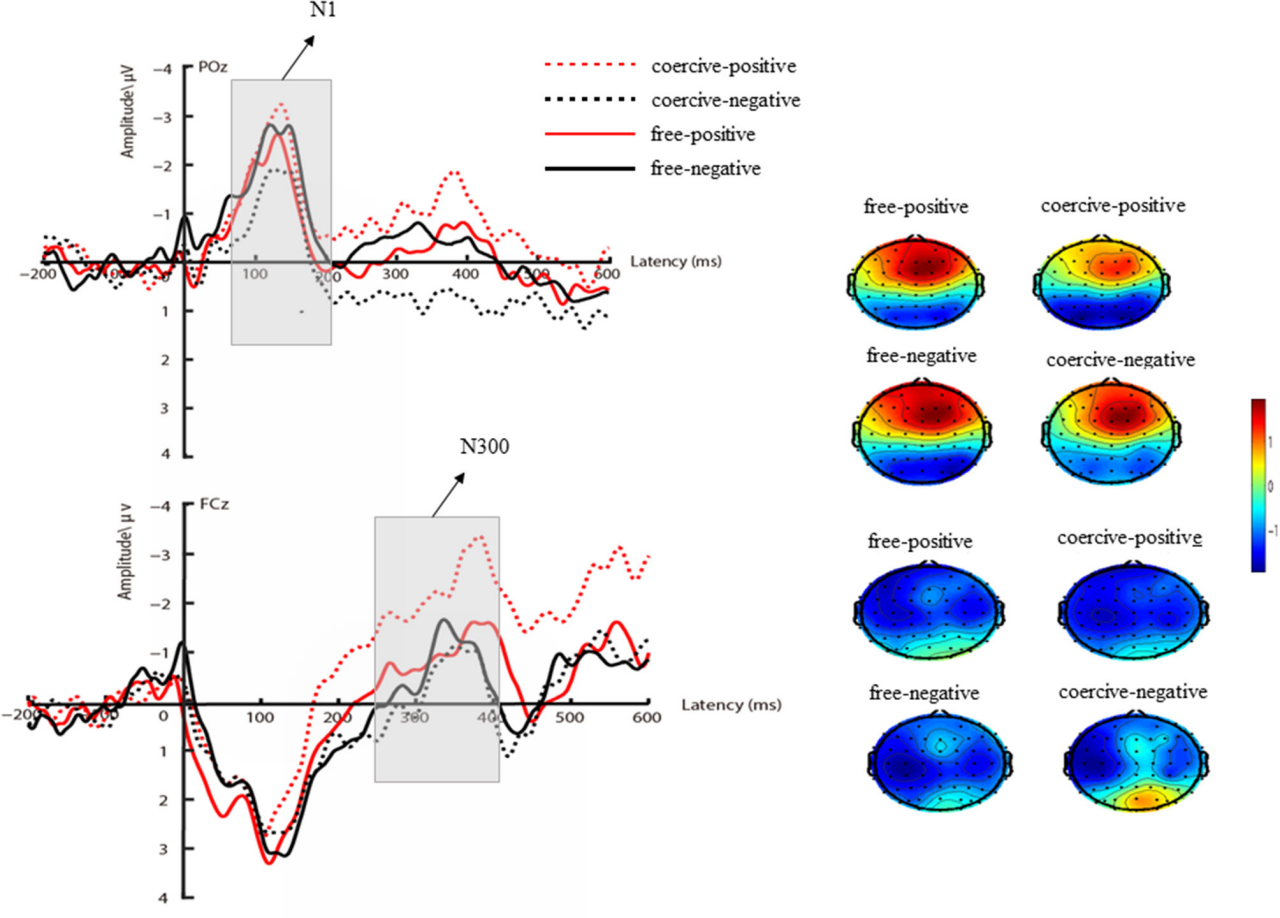
**(Left)** Averaged ERPs for the different conditions (coercive-positive, coercive-negative, free-positive, and free-negative). **(Right)** Scalp topography of N1 and N300 under each condition.

The results indicated that voluntary action was not significant, *F*_(1,26)_ = 0.002, *p* = 0.96. In addition, the main effect of outcome valence was not significant, *F*_(1,26)_ = 0.82, *p* = 0.37, and $\eta_{p}^{2}$ = 0.03. However, the interaction between outcome valence and voluntary action was significant, *F*_(1,26)_ = 9.04, *p* < 0.01, and $\eta_{p}^{2}$ = 0.26. Further analyses revealed that the positive tones elicited larger N1 amplitude (*M* = −1.90 μV, *SD* = 0.50 μV) compared with negative tones (*M* = −1.10 μV, *SD* = 0.44 μV, and *p* < 0.05) in the coercive condition. The N1 component induced by negative (*M* = −1.73 μV, SD = 0.46 μV) and positive tones (*M* = −1.33 μV, *SD* = 0.41 μV, and *p* = 0.18) had no significant difference in the free condition. There were also no significant differences in other main effects and interactions (*ps* > 0.05; see Figure 3).

#### 3.3.2. N300

The results indicated that the main effect of outcome valence was significant, *F*_(1,26)_ = 5.23, *p* < 0.05, and $\eta_{p}^{2}$ = 0.17. Compared with negative tones (*M* = −1.04 μV, *SD* = 0.42 μV), positive tones elicited more negative N300 (*M* = −1.89 μV, *SD* = 0.54 μV). The main effect of voluntary action was not significant, *F*_(1,26)_ = 0.44, *p* = 0.51, and $\eta_{p}^{2}$ = 0.02.

**Figure 4:**
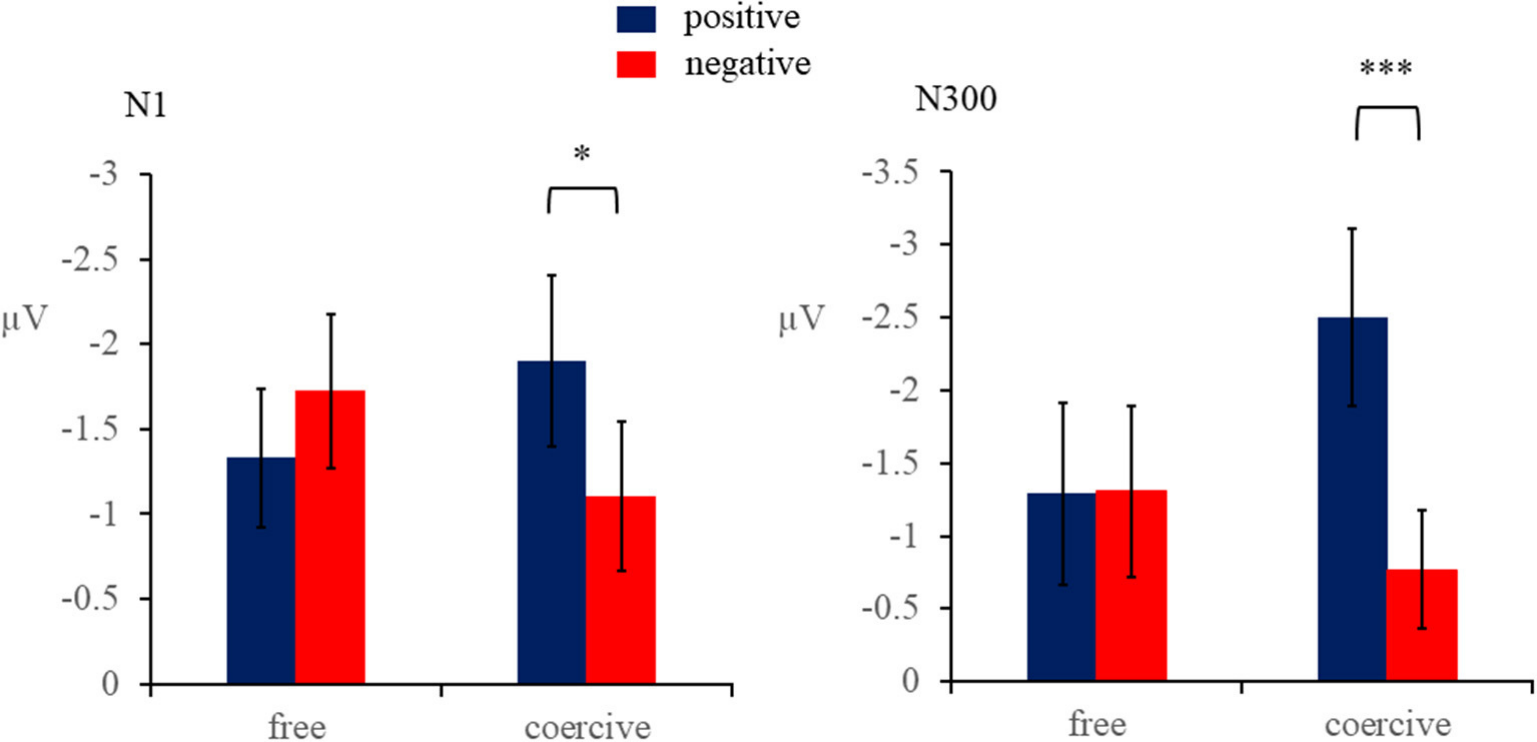
Mean amplitudes of N1 (70–200 ms) and N300 (250–400 ms). *p < 0.05, ***p < 0.001.

The interaction between outcome valence and voluntary action was significant, *F*_(1,26)_ = 8.76, *p* < 0.01, and $\eta_{p}^{2}$ = 0.25. Further analyses revealed that the N300 component induced by positive tones (*M* = −2.50 μV, *SD* = 0.61 μV) was more negative than that by negative tones (*M* = −0.77 μV, *SD* = 0.41 μV, and *p* < 0.001) in the coercive condition. The N300 component induced by positive tones (*M* = −1.29 μV, *SD* = 0.63 μV) and negative tones (*M* = −1.31 μV, *SD* = 0.59 μV, and *p* = 0.97) was not significantly different in the free condition. There were no significant differences in other main effects and interactions, *ps* > 0.05 (see Figure 4).

We further adopted regression analysis to control the potential influence of electric stimuli proportion on N1 and N300. The results showed that the prediction effect of electric stimuli proportion on N1 and N300 was not significant in each condition, *ps* > 0.05, indicating that the proportion of electric stimuli did not influence the N1 and N300.

### 3.4. Correlation between ERP and the sense of agency

Considering that the implicit sense of agency and tone-induced ERP were influenced by the interaction of voluntary action and outcome valence, both reflecting the implicit processing across different dimensions of the sense of agency, there could be a correlation between the implicit sense of agency and tone-induced ERP. Therefore, the differences between positive and negative tones were separately calculated under coercive and free conditions, which were conducted using Pearson correlation analysis between ERP and temporal intervals. The results revealed that under the coercive condition, there was no significant correlation between the perceived temporal interval and the tone-induced N1 component (*r* = −0.29, *p* = 0.14), and no significant correlation was observed between the perceived temporal interval and the tone-induced N300 component (*r* = −0.32, *p* = 0.11). However, the correlations reached a moderate level, suggesting that the lack of significant correlation may be owing to a small sample size. Under the free condition, there was no significant correlation between the perceived temporal interval and the tone-induced N1 component (*r* = 0.03, *p* = 0.88), and no significant correlation was observed between the perceived temporal interval and the N300 component (*r* = 0.11, *p* = 0.59).

## 4. Discussion

This study investigated the embodied psychological mechanisms through which individuals control their behavior and consequent outcomes in unethical contexts. More specifically, this study examined the moderating effects of outcome valence on the sense of agency when participants were given the freedom or coerced into administering electric stimuli to strangers. The results showed that the estimated interval of positive tones was smaller than that of negative tones when obeying commands, which induced more negative N1 and N300, indicating a stronger implicit sense of agency. Regarding the explicit sense of agency, our study only found that participants in the free condition reported a higher feeling of control compared with those in the coercive one. Our results indicated that the sense of agency could arise from the integration of predictive cues and retrospective reasoning when individuals follow orders or make a free choice regarding whether to administer electric stimuli to others. The study indicates the implications of using positive outcome feedback and free choice as significant means of intervention for the abnormal sense of agency.

### 4.1. Outcome valence moderates the effect of voluntary action on the implicit sense of agency

As predicted, our study found that when obeying commands, the estimated temporal interval between keypress actions and consequent tones was smaller in the coercive-positive condition than in the coercive-negative condition, which supported our hypothesis that positive tonal outcomes could enhance individuals' experience of the sense of agency. This finding fits with previous studies relating to the implicit sense of agency. Studies have demonstrated that individuals report larger temporal intervals between self-actions and consequent outcomes when obeying orders to administer electric shocks to strangers, indicating a decrease in the implicit sense of agency (Caspar et al., 2016, 2017, 2018). However, when hearing positive tonal outcomes, individuals could experience stronger moral conflicts in the coercive condition, the implicit sense of agency was higher under severe moral conflict than under moderate moral conflict (Moretto et al., 2011), retrospectively enhancing the causal relationship between actions and subsequent outcomes. This result extended previous research and obtained corresponding support for the ERP results.

Regarding the ERP results, we observed that in the coercive condition, the N1 component induced by positive tones was more negative than by negative ones, indicating the occurrence of sensory enhancement rather than sensory attenuation. This finding contradicted previous findings that demonstrated smaller N1 amplitudes associated with self-initiated actions. For example, numerous studies have shown that self-generated auditory stimuli (e.g., tone) or visual stimuli (i.e., pictures) elicit a weaker N1 compared with those generated by others, indicating sensory attenuation (Schafer and Marcus, 1973; Gentsch et al., 2015). In addition, sensory attenuation was observed regardless of whether the tones were predictable or not for self-initiated actions (Bäß et al., 2008). However, researchers have found that the N1 component induced by neutral tones is more negative under the free condition than under the coercive condition (Caspar et al., 2016). This finding provides evidence that the N1 component reflects the causal relationship between behavior and outcome (Caspar et al., 2016). Our study, using the consistent experimental paradigm employed by Caspar et al. (2016), supported the aforementioned finding and expanded the scope of existing studies. Based on the preceding discussion, we suggest that individuals in the coercive-positive condition could be unable to ignore their role as the agent inflicting harm on others during the early stage of cognitive processing, which could lead to sensory enhancement.

Furthermore, during the later stages of cognitive processing, we observed that the N300 component was more negative in the coercive-positive condition compared with the coercive-negative one. This finding extended previous research. The N300 component is linked to the affective evaluation of stimuli (Carretié and Iglesias, 1995; Rossignol et al., 2005; Ruz et al., 2013), which is supposed to reflect the depth of affective processing or the affective significance of stimuli rather than the physical characteristics (Rossignol et al., 2005; Ruz et al., 2013). The self-generated visual stimuli (i.e., pictures) elicit more negative N300 compared with externally initiated stimuli, and self-generated negative outcomes evoke more negative N300 compared with self-generated positive outcomes during the later stages of cognitive processing (Gentsch et al., 2015). Consequently, positive outcomes induced more negative N300 in the coercive condition, which might affect the self-serving bias, leading to an increased implicit sense of agency in morally unethical situations. Furthermore, it would be valuable for future research to investigate the role of self-serving bias on the sense of agency in morally unethical situations.

In addition, although our study found insignificant correlations between the differences in N1 or N300 amplitudes induced by positive and negative tones and the differences in the implicit sense of agency under the coercive condition, the correlation coefficients reached a moderate level. We speculated that the lack of significant correlation between the implicit sense of agency and ERP might be owing to a small sample size. Further research is needed to investigate the correlation between behavior and tone-induced ERP results.

Overall, the N1 and N300 components elicited by positive tones were more negative compared with negative tones in the coercive condition, and participants perceived a shorter temporal interval between self-actions and consequent outcomes. These findings suggested that individuals were unable to implicitly disregard the fact that they themselves were subjected to electric shocks, which indicated that the implicit sense of agency integrates information from both predictive cues and retrospective reasoning in different situations.

### 4.2. The separate mechanisms underlying implicit and explicit senses of agency

Importantly, this study revealed the separate mechanism underlying the implicit and explicit senses of agency, which revealed that the explicit sense of agency was only influenced by voluntary action, and outcome valence moderated the influence of voluntary action on the implicit sense of agency. The results were partially supported by previous research (Dewey and Knoblich, 2014; Majchrowicz and Wierzchoń, 2018). For example, the correlation between the implicit and explicit senses of agency is not significant, suggesting that the mechanisms underlying implicit and explicit senses of agency are different at least to some extent (Zopf et al., 2018). In addition, researchers have discovered that the two implicit measures of sense of agency (interval estimation and sensory attenuation) do not show a significant correlation, and no significant correlation is observed between the implicit and explicit senses of agency (Dewey and Knoblich, 2014; Majchrowicz and Wierzchoń, 2018), which suggests that the sense of agency could involve different processing mechanisms.

The multilevel model proposes that the sense of agency is composed of the “feeling of agency,” the “judgment of agency,” and the “meta-representation of agency” (Synofzik et al., 2008b). Specifically, “feeling of agency” arises from perceptual representations of actions and is a pre-reflective, implicit, non-conceptual sense of agency, relying on the match between the anticipated outcome and the actual sensory feedback. “Judgment of agency” is a reflective, explicit, conceptual judgment that predominantly relies on the awareness and inference of the causal relationship between actions and outcomes (Bu et al., 2022). “Meta-Representation of agency” is derived from moral responsibility judgments made at the level of attributing actions. Based on this, the implicit sense of agency reflects the unconscious level of processing (Synofzik et al., 2008a). Contrastingly, the explicit sense of agency can be influenced by cognitive biases, notably social desirability (e.g., avoiding blame or punishment) (Bu et al., 2022). Individuals could consciously tend to deny the causal connection to the electric shock initiated by themselves in the coercive condition, possibly due to the influence of social desirability (Caspar et al., 2016, 2018). Therefore, the sense of agency is clearly influenced by implicit, pre-reflective bottom-up processing and explicit, reflective, conscious top-down processing (Bu et al., 2022), indicating the different processing mechanisms of the implicit and explicit senses of agency, thus resulting in the observed separation.

### 4.3. Limitations and future directions

This study has implications for individuals experiencing the abnormal sense of agency, such as passive symptoms of schizophrenia and delusions of control, and could contribute to potential interventions or treatments. In future, free choice and positive outcome feedback can be effective means of pushing for improvements in the abnormal experience of the sense of agency. In addition, outcome valence was found to moderate the effect of voluntary action on implicit senses of agency. In future, the influence of the unpredictability of outcome valence on the sense of agency can be further explored.

Indeed, our study had some limitations. First, personality traits (e.g., feeling of control) related to the sense of agency were not considered. Although this study used a within-subject experimental design with minimal impact from trait differences, the trait of the feeling of control could be a potential variable that interferes with explicit subjective ratings. Future research is needed to explore the impact of personality traits on the explicit sense of agency. Second, our research exclusively included university students, which comprises a relatively homogenous group. Therefore, more caution is needed when generalizing the results to a wider group.

## 5. Conclusion

In summary, this study showed that the influence of voluntary action on the implicit sense of agency was moderated by outcome valence. Compared with the negative outcome, the implicit sense of agency under the positive outcome was higher in the coercive condition and induced more negative N1 and N300 components. In addition, the explicit sense of agency was only affected by voluntary action, which showed that the feeling of control was lower when obeying orders. This study showed that implicit and explicit sense of agency involved different processing mechanisms of predictive cues and retrospective reasoning.

## Data availability statement

The raw data supporting the conclusions of this article will be made available by the authors, without undue reservation.

## Ethics statement

The studies involving human participants were reviewed and approved by the Ethics Committee of Hunan Normal University. The patients/participants provided their written informed consent to participate in this study.

## Author contributions

NN and YZ designed experiment and wrote the manuscript. NN, YW, DY, and ML carried out the experiments. NN, WF, and HL analyzed the data. All authors edited and approved the manuscript.
